# Bioassay-guided isolation of leishmanicidal cucurbitacins from *Momordica charantia*

**DOI:** 10.3389/fphar.2024.1390715

**Published:** 2024-07-11

**Authors:** Maria Carolina Silva Marques, Nídia Cristiane Yoshida, Eduardo Caio Torres-Santos, Fernanda Rodrigues Garcez, Walmir Silva Garcez

**Affiliations:** ^1^ Laboratory of Microbiology, Institute of Biosciences, Federal University of Mato Grosso do Sul (UFMS), Campo Grande, Brazil; ^2^ Laboratory of Bioactive Natural Products Research (PRONABio), Institute of Chemistry, Federal University of Mato Grosso do Sul (UFMS), Campo Grande, Brazil; ^3^ Laboratory of Trypanosomatid Biochemistry, Oswaldo Cruz Foundation (FIOCRUZ), Rio de Janeiro, Brazil

**Keywords:** *Momordica charantia*, Cucurbitaceae, leishmanicidal activity, *Leishmania amazonensis*, neglected tropical disease, cucurbitacin

## Abstract

**Introduction:**

Leishmaniasis, a neglected tropical parasitic disease, is regarded as a major public health problem worldwide. The first-line drugs for leishmaniasis suffer from limitations related to toxicity and the development of resistance in certain parasitic strains. Therefore, the discovery of alternative treatments for leishmaniasis is imperative, and natural products represent a valuable source of potential therapeutic agents.

**Methods:**

The present study aimed at finding new potential antileishmanial agents from the aerial parts of the medicinal plant *Momordica charantia*. This study was based on bioassay-guided fractionation of the *M. charantia* extract against promastigotes and amastigotes of *Leishmania* (*Leishmania*) *amazonensis*. The cytotoxicity of the extract, fractions, and isolated compounds were evaluated against peritoneal murine macrophages by employing the MTT assay for assessing cell metabolic activity.

**Results:**

Antileishmanial assay-guided fractionation of the *M. charantia* extract led to the bioactive cucurbitacin-enriched fraction and the isolation of four bioactive cucurbitacin-type triterpenoids, which exhibited significant antileishmanial activity, with IC_50_ values between 2.11 and 3.25 μg.mL^−1^ against promastigote and amastigote forms, low toxicity and selectivity indexes ranging from 8.5 to 17.2.

**Conclusion:**

Our findings demonstrate that the fractions and cucurbitacin-type triterpenoids obtained from the aerial parts of *M. charantia* are promising natural leishmanicidal candidates.

## 1 Introduction

Leishmaniasis, a neglected tropical parasitic disease, is considered a major public health problem worldwide. This ailment is caused by unicellular protozoan organisms of the *Leishmania* genus, which belong to the Trypanosomatidae family (Mishra et al., 2009). Leishmaniasis accounts for an estimated 700,000 to a million new cases worldwide each year. In 2020, over 90% of the new cases of visceral leishmaniasis and 85% of cutaneous leishmaniasis cases reported to the World Health Organization (WHO) originated from 10 countries (Medenica et al., 2023). In Brazil, *Leishmania* (*Viannia) braziliensis* and *L*. (*Leishmania*) *amazonensis* species are widely distributed. Among these, *L*. (*Leishmania*) *amazonensis* is known to cause a broad spectrum of clinical diseases, ranging from localized cutaneous leishmaniasis to visceral leishmaniasis and mucocutaneous forms (Anversa et al., 2018).

Pentavalent antimonials are the first-line drugs for the treatment of all types of leishmaniasis (Polonio and Efferth, 2008). However, despite the development of an array of chemotherapeutic agents, none of the drugs are entirely free from side effects; a number of these suffer limitations related to unaffordability, difficulty of administration, and toxicity. In addition, antileishmanial activity seems to be dependent on the evaluated *Leishmania* species (Barrett and Croft, 2012). The development of resistance in some parasitic strains is an additional concern (Padma, 2013). These limitations, combined with the absence of an effective vaccine, demonstrate the urgency for the development of new alternatives for the treatment of leishmaniasis (Sen and Chatterjee, 2011).

In recognition of the impact of leishmaniasis worldwide, WHO has proposed that the diffusion of the disease should be controlled and evaluation of the efficacy of new medicines should be promoted as one of the strategies of the control program (World Health Organization WHO, 2010). Plant extracts and plant-derived compounds have been used to treat leishmaniasis and represent potential sources of new medicinal agents and antileishmanial drugs (Newman and Cragg, 2020).

As part of our ongoing investigation on the use of Cerrado plants as a source of bioactive molecules, ethanol extracts from 14 species belonging to 12 different families have been analyzed for their activity against promastigote forms of *L. amazonensis* (Marques et al., 2013). It was determined that the crude ethanol extract obtained from the aerial parts of *Momordica charantia* L. (Cucurbitaceae) showed a promising activity profile, with an IC_50_ of 6.25 μg.mL^−1^.

*Momordica charantia* is used in traditional medicine as an antidiabetic, antihelmintic, and antimalarial, and to treat leprosy, skin diseases and scabies, and it has been described as a producer of biologically active phytochemicals such as triterpenoids, alkaloids, and steroids (Grover and Yadav, 2004). Several studies regarding the biological activities of *M. charantia* have detailed its use as an adjuvant for the treatment of diabetes to reduce glucose levels (Rathi et al., 2002), as well as oxidative stress (Sathishsekar and Subramanian, 2005), further, it has been used as an antiviral therapy for HIV infection (Cunnick et al., 1990), as a cytostatic agent in certain types of cancers (Ganguly et al., 2000), and as an antiprotozoal agent against *Trypanosoma* and *Plasmodium* spp. (Munoz et al., 2000; Kohler et al., 2002). Previous investigations on the leishmanicidal potential of *M. charantia* were performed with the ethanolic extract of the leaves, which exhibited IC_50_ values of 58.4 and 500 μg.mL^−1^ against promastigote forms of *L. amazonensis* (García et al., 2012) and *L. brasiliensis* (Santos et al., 2013), respectively. The ethanolic extract of the green fruits of *M. charantia* proved to be active against *Leishmania donovani* promastigotes, and further investigation led to the identification of momordicatin [4-(*O*-carboethoxyphenyl) butanol] as the active compound (Gupta et al., 2010).

In the present study, by using a bioassay-guided approach, we assessed the activity and toxicity of the extract, phases, and isolated compounds obtained from *M. charantia*, against extracellular promastigote and intracellular amastigote stages of *L. (Leishmania*) *amazonensis*.

## 2 Materials and methods

### 2.1 Plant extract preparation

*Momordica charantia* L. (Cucurbitaceae) samples were collected in Corumbá (19°34′37″S, 57°00′42″W), Mato Grosso do Sul, Brazil, in December 2007. The plant material was identified by Dr. Arnildo Pott and M. Sc. Ubirazilda Maria Rezende (CGMS Herbarium, Universidade Federal de Mato Grosso do Sul, Brazil), where a voucher specimen (No. 19894) is deposited. The research with this plant material was registered in the National System of Genetic Resource Management and Associated Traditional Knowledge (SisGen) at number A685E68. The aerial parts (leaves and stems) of *M. charantia* (150 g) were dried, triturated and extracted with 95% EtOH (×5, 750 mL) for 5 days at room temperature. The resulting extracts were combined and the solvent was removed under reduced pressure. The residue obtained (53.1 g) was stored at −18°C.

### 2.2 Antileishmanial bioassay-guided isolation of cucurbitacins

The ethanol extract of *M. charantia* (EEMc), which exhibited antileishmanial activity (IC_50_ = 6.25 μg.mL^−1^), was re-suspended in MeOH-H_2_O (8:2) and successively partitioned with hexanes and EtOAc (Supplementary Figure S1). The resulting phases were tested for antileishmanial activity, wherein the EtOAc phase was found to possess a superior activity profile (IC_50_ = 6.17 μg.mL^−1^). The EtOAc phase was chromatographed on a silica gel 70–230 mesh column, using step gradient elution with hexanes-EtOAc to yield 14 fractions (F1−F14). Fraction F7 showed the highest antileishmanial activity (IC_50_ = 3.02 μg.mL^−1^), and an aliquot of this fraction (0.50 g) was further chromatographed on a Sephadex LH-20 column eluted with CHCl_3_, to give 11 subfractions (A1−A11), of which A1 (44.8 mg), A3 (92.3 mg), and A5 (17.6 mg) exhibited antileishmanial activity. Fraction A1 was re-chromatographed on a silica gel 200–400 mesh column [hexanes-EtOAc (6:4)] to afford 11 subfractions (A1.1-A1.11), of which A1.3 yielded 25-methoxy-3,7-dihydroxycucurbita-5,23(*E*)-dien-19-al (**1,** 10.1 mg). 3,7,25-trihydroxycucurbita-5,23(*E*)-dien-19-al (**2**, 90.0 mg), was isolated from subfraction A3, after reversed-phase semi-preparative HPLC (CH_3_CN-H_2_O, 50→100%). Fraction A5 was re-chromatographed on a silica gel 200–400 mesh column [hexanes-EtOAc-MeOH (6:4:0.5)] to afford nine subfractions (A5.1-A5.9). 19(*R*),25-dimethoxy-5-19-epoxycucurbita-6,23(*E*)-dien-3-ol (**3**, 2.1 mg), was obtained from subfraction A5.2, while 19(*R*)-methoxy-5-19-epoxycucurbita-6,23(*E*)-dien-3,25-diol (**4**, 1.7 mg) was isolated from subfraction A5.3, after preparative TLC on silica gel PF_254_, solvent system hexanes-EtOAc-MeOH (6:2:0.5). Compounds **1–4** were identified by 1D and 2D NMR spectroscopy recorded at 300 MHz for ^1^H and 75 MHz for ^13^C NMR, on a Bruker DPX-300 spectrometer, and by comparison with literature data.

### 2.3 HPLC analysis

HPLC analysis was performed on a Shimadzu system (Shimadzu Corporation, Kyoto, Japan) equipped with LC-6AD pumps, a SIL-20A autosampler, SPDV-6AV UV/VIS detector, CBM-20A communications module and LC Solution 1.24 SP2 software to record and process the data. Acetonitrile (CH_3_CN) was HPLC grade (Merck, Darmstadt, Germany), and H_2_O was purified using a Milli-Q system (Millipore, Bedford, MA, United States). The analyses were performed using a Phenomenex Luna^®^ C18 column (5 μm, 100 Å, LC column 4.6 × 250 mm). Cucurbitacins **1–4** were eluted with an isocratic mobile phase system consisting of CH_3_CN-H_2_O (70:30) and detected at 210 nm. The extracts were completely dissolved in CH_3_CN, filtered (0.22 µm pore size, Merck), and analyzed at room temperature (25°C), at a flow rate of 1 mL.min^−1^ and an injection volume of 10 µL. Peaks were identified by comparison of their retention times (t_R_) (**1** = 7.12 min; **2** = 9.42 min; **3 + 4** = 19.29 min) and co-injection with the isolated standards (Supplementary Figure S10). The identities and purities of the compounds were confirmed by NMR spectroscopy.

### 2.4 *L*. (*Leishmania*) *amazonensis* culture

The *L*. (*L*.) *amazonensis* strain MHOM/BR/77/LTB0016 was kept at 26°C in Schneider’s insect medium (Sigma-Aldrich, St. Louis, United States), at pH 6.9, and supplemented with 10% heat-inactivated fetal calf serum, 100 mg.mL^−1^ of streptomycin, and 100 IU/mL of penicillin. Parasites were maintained in culture until the 10th passage and new parasites were routinely isolated from infected lesions of BALB/c mice.

### 2.5 Determination of leishmanicidal activity *in vitro*

#### 2.5.1 Antipromastigote activity

The effects of the crude extract, fractions, and isolated compounds on the viability of the extracellular forms of *L. amazonensis* were determined using the thiazolyl blue tetrazolium bromide (MTT) assay (Sigma-Aldrich, St. Louis, United States). A stock solution of testing materials and pentamidine was prepared in DMSO (Sigma Aldrich). The maximum solvent concentration used in the assay was 0.5% in a final volume per well of 200 µL. Untreated infected cells and pentamidine isethionate (3.125–200 μM.mL^−1^) were used as a negative and positive control, respectively. The logarithmic phase of growth was maintained, and the final concentration of parasites was adjusted to 1 × 10^6^ promastigotes.mL^−1^. The cells were transferred to 96-well plates, incubated for 72 h, and kept at 27°C with the testing material (extract and fractions: 1.56–50 μg.mL^−1^; pure compounds: 3.125–200 μM.mL^−1^). Following incubation, 22 µL of MTT solution (5 μg.mL^−1^) was added to each well, and samples were incubated for 2 h, followed by the addition of 80 µL of DMSO. The optical density was measured at 570 nm on a µQuant system (Bio-Tek Instruments, Winooski, United States). The results were expressed as the mean ± standard deviation (SD) of the IC_50_ of three independent experiments done in triplicate. Compounds with IC_50_ ≤ 50 μg.mL^−1^ were considered active.

#### 2.5.2 Anti-amastigote activity

For the evaluation of activity against intracellular amastigotes, murine peritoneal macrophage cells were obtained by peritoneal washing with 5 mL of iced Roswell Park Memorial Institute–1640 (RPMI-1640) medium (Sigma). The peritoneal fluid was adjusted to a concentration of 2 × 10^6^ macrophages. mL^−1^, plated (0.4 mL/well) in Lab-Tek 8-chambers slides (Nunc, Roskilde, Denmark) and incubated for 1 h at 37°C and 5% CO_2_. The cultures were washed with phosphate-buffered saline (PBS) at 37°C to remove the non-adherent cells. The adhered cells were incubated with *L. amazonensis* promastigotes in the ratio of 3:1 (0.4 mL/well) at 37°C and 5% CO_2_. After 4 h, the chambers were washed again to remove free parasites. For the assay against amastigote forms, monolayers of the cells containing the intracellular parasites were incubated with the test compounds and the controls at a range of concentrations (1.5625–50 μg.mL^−1^) for 72 h at 37°C and 5% CO_2_. After the incubation period, the cells were washed with phosphate buffer saline (0.02 M, pH 7.2), fixed with methanol and stained with Giemsa, to count live amastigotes in macrophage by bright-field microscopy. The anti-amastigote activities of the compounds were determined microscopically by counting the number of amastigotes and examining at least 100 infected macrophages per experiment. The results were expressed as an infection index (% infected macrophage×number of amastigote/total number of macrophages) and IC_50_ values were calculated by plotting the infection index in amastigotes against drug concentrations tested by logarithmic nonlinear regression analysis, using GraphPad Prism software version 5.0. Due to limited quantities of **3** and **4**, their anti-amastigote activity and cytotoxicity against mammalian cells could not be evaluated, and accordingly, their selectivity indexes. The results were expressed as the mean ± standard deviation (SD) of the IC_50_ of three independent experiments done in triplicate.

### 2.6 *In vitro* cytotoxic concentration (CC_50_) against mammalian cells

The cytotoxicity of the compounds was determined using murine macrophages (2 × 10^6^ in 200 µL) of BALB/c mice incubated with various compound concentrations for 72 at 37°C and 5% CO_2_. The effect on macrophage viability was assessed using the MTT assay. The concentration of extracts/compounds that caused 50% macrophage cytotoxicity (CC_50_) was determined by linear regression analysis using GraphPad Prism software, version 5.0. The selectivity index (SI) of the extracts/compounds was determined using the following equation: SI = CC_50_ against mammalian cells/IC_50_ against *L. amazonensis* amastigotes.

### 2.7 Statistical analyses

The *in vitro* antileishmanial activity and cytotoxicity were expressed as the IC_50_ and CC_50_, respectively, by a non-linear regression curve calculated using the statistical GraphPad Prism software version 5.0, with a confidence interval of 95%.

## 3 Results

### 3.1 Isolation and characterization of cucurbitacins

**FIGURE 1:**
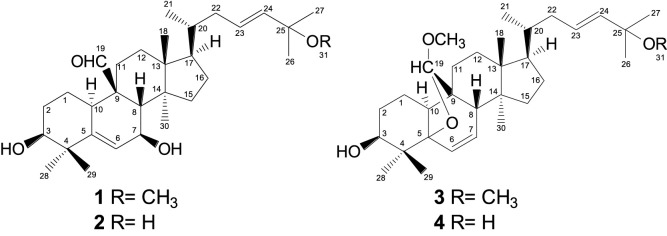
Structures of the isolated compounds obtained from the ethanol extract of aerial parts of *Momordica charantia*.

A bioassay-guided fractionation of the bioactive fraction F7 led to the isolation and characterization of four cucurbitacins: 25-methoxy-3,7-dihydroxycucurbita-5,23(*E)*-dien-19-al (**1**), 3,7,25-trihydroxycucurbita-5,23(*E*)-dien-19-al (**2**), 19(*R*),25-dimethoxy-5-19-epoxycucurbita-6,23(*E*)-dien-3-ol (**3**), and 19(*R*)-methoxy-5-19-epoxycucurbita-6,23(*E*)-dien-3,25-diol (**4**) (Figure 1). Their structures were established based on NMR experiments (Supplementary Table S1). Tetracyclic triterpenes with cucurbitane-type rearranged skeletons, such as compounds **1–4**, have been previously isolated from the leaves and fruits of *M. charantia* collected in Japan and Nigeria. The NMR data obtained for these compounds in our study were consistent with those reported in the literature (Fatope et al., 1990; Kimura et al., 2005). Additionally, cucurbitacins **1–4** have been identified in the leaves of *Momordica foetida* collected in South Africa (Mulholland et al., 1997).

### 3.2 HPLC profiles

**FIGURE 2:**
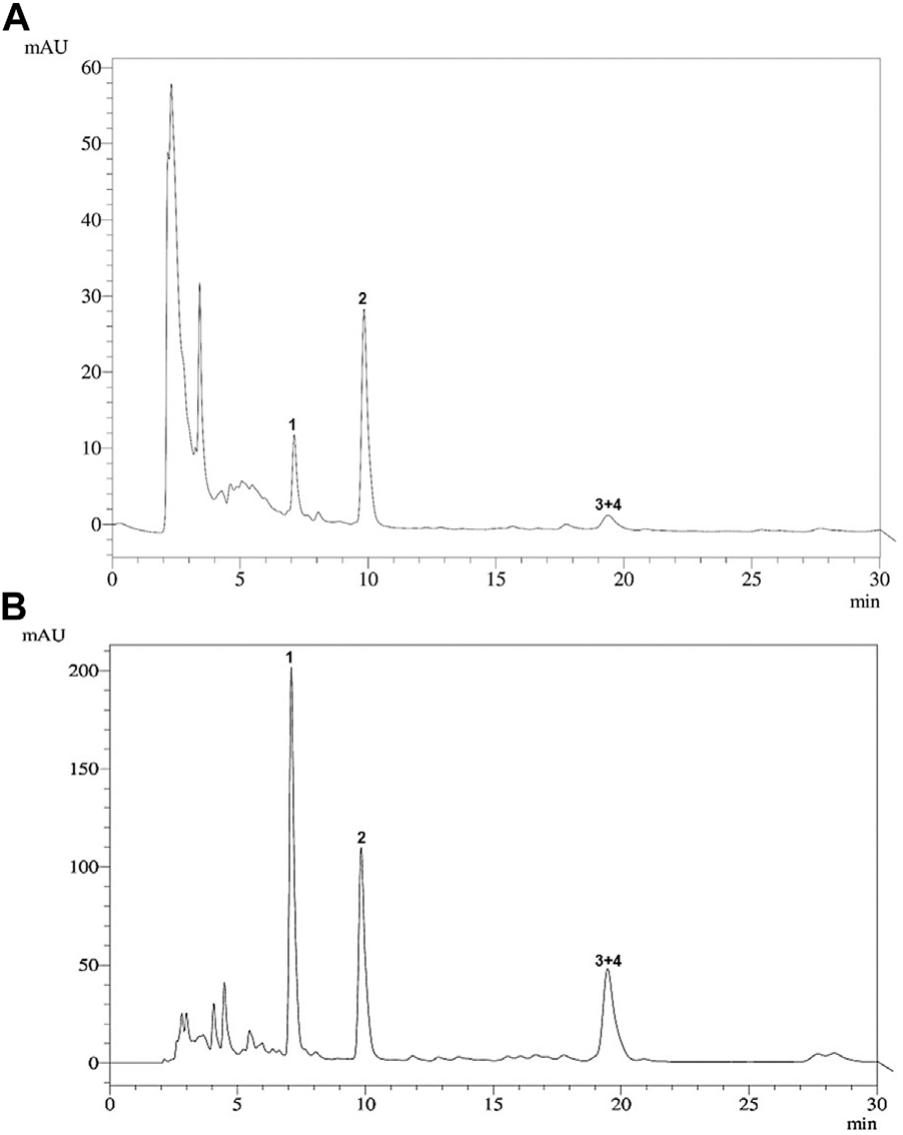
HPLC profile of **(A)** the ethanol extract of the aerial parts and **(B)** cucurbitacin-enriched fraction F7 of *Momordica charantia*. **1**: 25-methoxy-3, 7-dihydroxycucurbita-5,23(*E*)-dien-19-al (7.12 min); **2**: 3,7,25-trihydroxycucurbita-5,23(*E*)-dien-19-al (9.42 min); **3 + 4**: 19(*R*),25-dimethoxy-5-19-epoxycucurbita-6,23(*E*)-dien-3-ol and 19(*R*)-methoxy-5-19-epoxycucurbita-6,23(*E*)-dien-3,25-diol (19.29 min).

The HPLC profiles of the ethanol extract and fraction F7 were similar, demonstrating that both contained a mixture of cucurbitacins **1–4** as the major components (Figure 2).

### 3.3 Antileishmanial activities

The antileishmanial activities of the extracts, fractions and cucurbitacins obtained from the aerial parts of *M. charantia* against promastigotes and amastigotes of *L*. (*L*.) *amazonensis* are reported in Table 1. The effects of the *M. charantia* ethanol extract and fractions on the promastigote forms of *L*. (*L*.) *amazonensis* were monitored for 72 h, and it was established that the ethanol extract and fraction F7 showed activity against the promastigote form of *L. amazonensis*, with IC_50_ values of 6.25 and 3.02 μg.mL^−1^, respectively. The isolated cucurbitacins **1–4** showed an activity profile against promastigotes with IC_50_ values ranging from 4.68 to 5.21 μg.mL^−1^ (Table 1).

**TABLE 1:** Antileishmanial activity of ethanol extract, fraction, and isolated compounds from the aerial parts of *Momordica charantia* against *Leishmania* (*L.*) *amazonensis*.

Plant material	Promastigotes IC_50_ µg.mL^−1^ (µM)	Amastigotes IC_50_ (µg.mL^−1^) (µM)	Cytotoxicity^a^ CC_50_ (µg.mL^−1^)	Selectivity index (SI)^b^
Ethanol extract	6.25 ± 0.03	5.90 ± 0.54	50.41 ± 0.41	8.5
Fraction F7	3.02 ± 0.04	2.11 ± 0.22	36.50 ± 1.14	17.2
1	5.21 (10.7) ± 0.08	3.25 (6.7) ± 0.58	29.03 (59.7) ± 4.03	8.9
2	4.79 (10.1) ± 0.06	3.13 (6.6) ± 0.84	>50 (102.9)	>15.9
3	4.68 (9.4) ± 0.05	Nd	Nd	Nd
4	5.05 (10.4) ± 0.12	Nd	Nd	Nd
Pentamidine isethionate*	1.63 (2.7) ± 0.17	0.65 (1.1) ± 0.10	2.90 (4.9) ± 0.10	4.5

Nd, not determined. *Control drug.

^a^ Concentration required for a 50% decrease of infected macrophages in treated vs. non treated cells.

^b^ SI, ratio CC_50(Macrophages)_/IC_50(Amastigotes)_.

## 4 Discussion

Promising results were obtained against amastigote forms of *L. amazonensis*, whereby fraction F7 exhibited the highest activity with an IC_50_ value of 2.11 μg.mL^−1^, followed by **1** and **2,** with IC_50_ values of 3.25 and 3.13 μg.mL^−1^, respectively. The ethanol extract was likewise active against amastigotes, exhibiting an IC_50_ of 5.9 μg.mL^−1^.

To date, a large number of cucurbitacins and cucurbitacin-derived compounds have been isolated from the Cucurbitaceae species. The structures of cucurbitacins **1–4** isolated from *M. charantia* have the same degree of oxygenation, a similar side chain at C-17 bearing hydroxyl or methoxy substituents, and oxygenated C-19 bearing aldehyde or acetal functional groups, as well as an oxygenated C-3 position. It has been suggested that these structural characteristics are important for antipromastigote activity of triterpenes (Filho et al., 2009).

The skeleton of the cucurbitacin-type tetracyclic triterpenes exhibits lipophilic behavior, particularly in free form, as is the case for **1–4**. The lipophilic characteristics of these compounds may promote the efficiency of parasite membrane transportation, increasing antiprotozoan activity (Basselin and Robert-Gero, 1998; Cargnin et al., 2013).

Interestingly, fraction F7 presented the highest antileishmanial activity, exceeding that of the isolated compounds **1–4**. The four cucurbitacins were found to be present as the major metabolites of fraction F7, as demonstrated by HPLC analysis (Figure 2). A similar aspect was observed for the cucurbitane-type triterpenoid-enriched fraction obtained from *Trichosanthes dioica*, which showed an inhibitory effect on the *in vitro* growth of *L. donovani* promastigotes (Bhattacharya et al., 2013). Although cucurbitacins have previously undergone evaluation for various biological activities (Hill and Connolly, 2017), studies supporting the antileishmanial activity of this class of secondary metabolites remain scarce.

When evaluated for their selective toxicity on mammalian macrophage cells, no significant cytotoxic effects on the viability of macrophages were observed for the ethanol extract, fraction F7, and **1** and **2**. Their CC_50_/IC_50_ ratios ranged from 8.5 to 17.2, indicating good selectivity indexes, which were higher than that obtained for the control pentamidine isethionate, one of the primary drugs employed against leishmaniasis (Table 1). According to Katsuno et al. (2015), for a compound to be considered with low cytotoxicity, the selectivity index (SI) must be ≥ 10.

## 5 Conclusion

By using a bioassay-guided fractionation approach, the extract, fractions, and four cucurbitacin-type triterpenoids, obtained from the aerial parts of the medicinal plant *M. charantia*, were revealed to exhibit significant leishmanicidal activity against *L. amazonensis*. The fraction enriched with cucurbitacin-type triterpenoids presented a superior activity profile to those of the isolated compounds. In addition, the tested cucurbitacins **1** and **2,** and the active fraction proved to be noncytotoxic to mammalian cells, thereby displaying good selectivity indexes. Further studies are being conducted to investigate the medicinal applicability of the cucurbitacins and the cucurbitacin-enriched fraction obtained from *M. charantia* as potential antileishmanial candidates and/or as an adjuvant for the treatment and control of leishmaniasis.

## Data Availability

The original contributions presented in the study are included in the article/Supplementary Material, further inquiries can be directed to the corresponding author.

## References

[B1] Anversa L. Tiburcio M. G. S. Richini-Pereira V. B. Ramirez L. E. (2018). Human leishmaniasis in Brazil: a general review. Rev. Assoc. Méd. Bras. 64 (3), 281–289. 10.1590/1806-9282.64.03.281 29641786

[B2] Barrett M. P. Croft S. L. (2012). Management of trypanosomiasis and leishmaniasis. Br. Med. Bull. 104 (1), 175–196. 10.1093/bmb/lds031 23137768 PMC3530408

[B3] Basselin M. Robert-Gero M. (1998). Alterations in membrane fluidity, lipid metabolism, mitochondrial activity, and lipophosphoglycan expression in pentamidine-resistant Leishmania. Parasitol. Res. 84, 78–83. 10.1007/s004360050361 9491432

[B4] Bhattacharya S. Biswas M. Haldar P. K. (2013). The triterpenoid fraction from *Trichosanthes dioica* root exhibits *in vitro* antileishmanial effect against *Leishmania donovani* promastigotes. Pharmacogn. Res. 5 (2), 109–112. 10.4103/0974-8490.110540 PMC3685758 23798885

[B5] Cargnin S. T. Vieira P. deB. Cibulski S. Cassel E. Vargas R. M. F. Montanha J. (2013). Anti-*Trichomonas vaginalis* activity of *Hypericum polyanthemum* extract obtained by supercritical fluid extraction and isolated compounds. Parasitol. Int. 62 (2), 112–117. 10.1016/j.parint.2012.10.006 23142570

[B6] Cunnick J. E. Sakamoto K. Chapes S. K. Fortner G. W. Takemoto D. J. (1990). Induction of tumor cytotoxic immune cells using a protein from the bitter-melon (*Momordica charantia*). Cell. Immunol. 126, 278–289. 10.1016/0008-8749(90)90321-H 2311123

[B7] Fatope M. O. Takeda Y. Yamashita H. Okabe H. Yamauchi T. (1990). New cucurbitane triterpenoids from *Momordica charantia* . J. Nat. Prod. 53 (6), 1491–1497. 10.1021/np50072a014

[B8] Filho A. A. S. Resende D. O. Fukui M. J. Santos F. F. Pauletti P. M. Cunha W. R. (2009). *In vitro* antileishmanial, antiplasmodial and cytotoxic activities of phenolics and triterpenoids from *Baccharis dracunculifolia* D. C. (Asteraceae). Fitoterapia 80 (8), 478–482. 10.1016/j.fitote.2009.06.007 19540316

[B9] Ganguly C. De S. Das S. (2000). Prevention of carcinogen-induced mouse skin papilloma by whole fruit aqueous extract of *Momordica charantia* . Eu. J. Cancer Prev. 9 (4), 283–288. 10.1097/00008469-200008000-00009 10958332

[B10] García M. Monzote L. Scull R. Herrera P. (2012). Activity of Cuban plants extracts against *Leishmania amazonensis* . ISRN Pharmacol. 2012, 104540. Article ID 104540. 10.5402/2012/104540 22530133 PMC3316957

[B11] Grover J. K. Yadav S. P. (2004). Pharmacological actions and potential uses of *Momordica charantia*: a review. J. Ethnopharmacol. 93 (1), 123–132. 10.1016/j.jep.2004.03.035 15182917

[B12] Gupta S. Raychaudhuri B. Banerjee S. Das B. Mukhopadhaya S. Datta S. C. (2010). Momordicatin purified from fruits of *Momordica charantia* is effective to act as a potent antileishmania agent. Parasitol. Int. 59 (2), 192–197. 10.1016/j.parint.2010.01.004 20132905

[B13] Hill R. A. Connolly J. D. (2017). Triterpenoids. Nat. Prod. Rep. 34, 90–122. 10.1039/c6np00094k 27813539

[B14] Katsuno K. Burrows J. N. Duncan K. Hooft van Huijsduijnen R. Kaneko T. Kita K. (2015). Hit and lead criteria in drug discovery for infectious diseases of the developing world. Nat. Rev. Drug Discov. 14 (11), 751–758. 10.1038/nrd4683 26435527

[B15] Kimura Y. Akihisa T. Yuasa N. Ukiya M. Suzuki T. Toriyama M. (2005). Cucurbitane-type triterpenoids from the fruit of *Momordica charantia* . J. Nat. Prod. 68 (5), 807–809. 10.1021/np040218p 15921438

[B16] Kohler I. Jenett-Siems K. Siems K. Hernandez M. A. Ibarra R. A. Berendsohn W. G. (2002). *In vitro* antiplasmodial investigation of medicinal plants from El Salvador. Z Naturforsch C J. Biosci. 57 (3-4), 277–281. 10.1515/znc-2002-3-413 12064726

[B17] Marques M. C. S. Hamerski L. Garcez F. R. Tieppo C. Vasconcelos M. Torres-Santos E. C. (2013). *In vitro* biological, screening and evaluation of free radical scavenging activities of medicinal plants from the Brazilian Cerrado. J. Med. Plants Res. 7 (15), 957–962. 10.5897/JMPR12.882

[B18] Medenica S. Miladinović-Tasić N. Stojanović N. M. Lakićević N. Rakočević B. (2023). Climate variables related to the incidence of human leishmaniosis in Montenegro in southeastern europe during seven decades (1945-2014). Int. J. Environ. Res. Public Health 20 (3), 1656. 10.3390/ijerph20031656 36767024 PMC9914530

[B19] Mishra B. B. Kale R. R. Singh R. K. Tiwari V. K. (2009). Alkaloids: future prospective to combat leishmaniasis. Fitoterapia 80 (2), 81–90. 10.1016/j.fitote.2008.10.009 19015012

[B20] Mulholland D. A. Sewram V. Osborne R. Pegel K. H. Connolly J. D. (1997). Cucurbitane triterpenoids from the leaves of *Momordica foetida* . Phytochemistry 45 (2), 391–395. 10.1016/s0031-9422(96)00814-x

[B21] Munoz V. Sauvain M. Bourdy G. Callapa J. Rojas I. Vargas L. (2000). The search for natural bioactive compounds through a multidisciplinary approach in Bolivia. Part II. Antimalarial activity of some plants used by Mosetene Indians. J. Ethnopharmacol. 69 (2), 139–155. 10.1016/S0378-8741(99)00096-3 10687870

[B22] Newman D. J. Cragg G. M. (2020). Natural products as sources of new drugs over the nearly four decades from 01/1981 to 09/2019. J. Nat. Prod. 83 (3), 770–803. 10.1021/acs.jnatprod.9b01285 32162523

[B23] Padma T. V. (2013). Leishmaniasis drug fails a fifth of patients: high failure rate of front-line therapy puts eradication campaign at risk. Nat. News. 10.1038/nature.2013.12553

[B24] Polonio T. Efferth T. (2008). Leishmaniasis: drug resistance and natural products (Review). Int. J. Mol. Med. 22 (3), 277–286. 10.3892/ijmm_00000020 18698485

[B25] Rathi S. S. Grover J. K. Vats V. (2002). The effect of *Momordica charantia* and *Mucuna pruriens* in experimental diabetes and their effect on key metabolic enzymes involved in carbohydrate metabolism. Phytother. Res. 16 (3), 236–243. 10.1002/ptr.842 12164268

[B26] Santos K. K. A. Rolón M. Veja C. Arias A. R. Costa J. G. M. Coutinho H. D. M. (2013). Atividade leishmanicida *in vitro* de *Eugenia uniflora* e *Momordica charantia* . Rev. Ciências Farm. Básica Apl. 34 (1), 47–50.

[B27] Sathishsekar D. Subramanian S. (2005). Antioxidant properties of *Momordica charantia* (bitter gourd) seeds on Streptozotocin induced diabetic rats. Asia Pac. J. Clin. Nutr. 14 (2), 153–158. 15927932

[B28] Sen R. Chatterjee M. (2011). Plant-derived therapeutics for the treatment of Leishmaniasis. Phytomedicine 18 (12), 1056–1069. 10.1016/j.phymed.2011.03.004 21596544

[B29] World Health Organization (WHO) (2010). Report of a meeting of the WHO Expert Committee on the control of leishmaniasis. Available at: http://whqlibdoc.who.int/trs/WHO_TRS_949_eng.pdf (Accessed April 18, 2016).

