# Supplementary material for: Bioassay-guided isolation of leishmanicidal cucurbitacins from Momordica charantia
Source: Front Pharmacol. 2024 Jul 11;15:1390715. doi: 10.3389/fphar.2024.1390715 (PMC11269121; doi:10.3389/fphar.2024.1390715)
Supplement: Supplementary file 1 [file DataSheet1.pdf]

Supplementary Material

Supplementary Figure 1. Flowchart showing the steps for the isolation and identification of cucurcubitacins from *Mamordica charantia*.

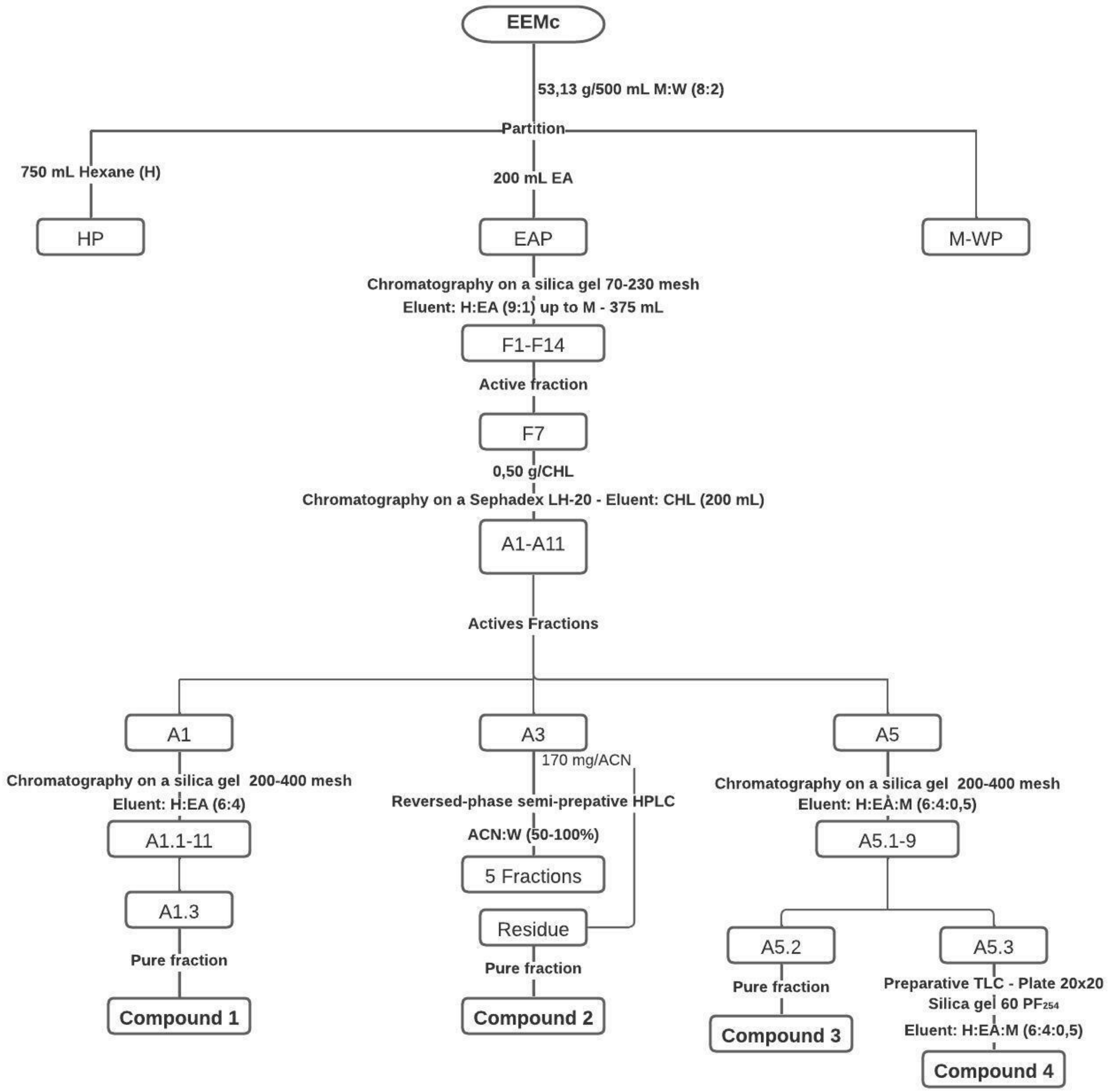

Abbreviations: EEMc - Ethanol extract of *Momordica charantia*; M - Methanol; W - Water; H - Hexane; EA - Ethyl acetate; HP - Hexane Fraction; EAP - Ethyl acetate Fraction; M-WP - Methanol-water Fraction; CHL - Chloroform; ACN - Acetonitrile.

Supplementary Figure 2. ^{1}H NMR spectrum of 25-methoxy-3β, 7β-dihydroxycucurbita-5,23(*E*)-dien-19-al (1) in acetone-d_6 (300 MHz)

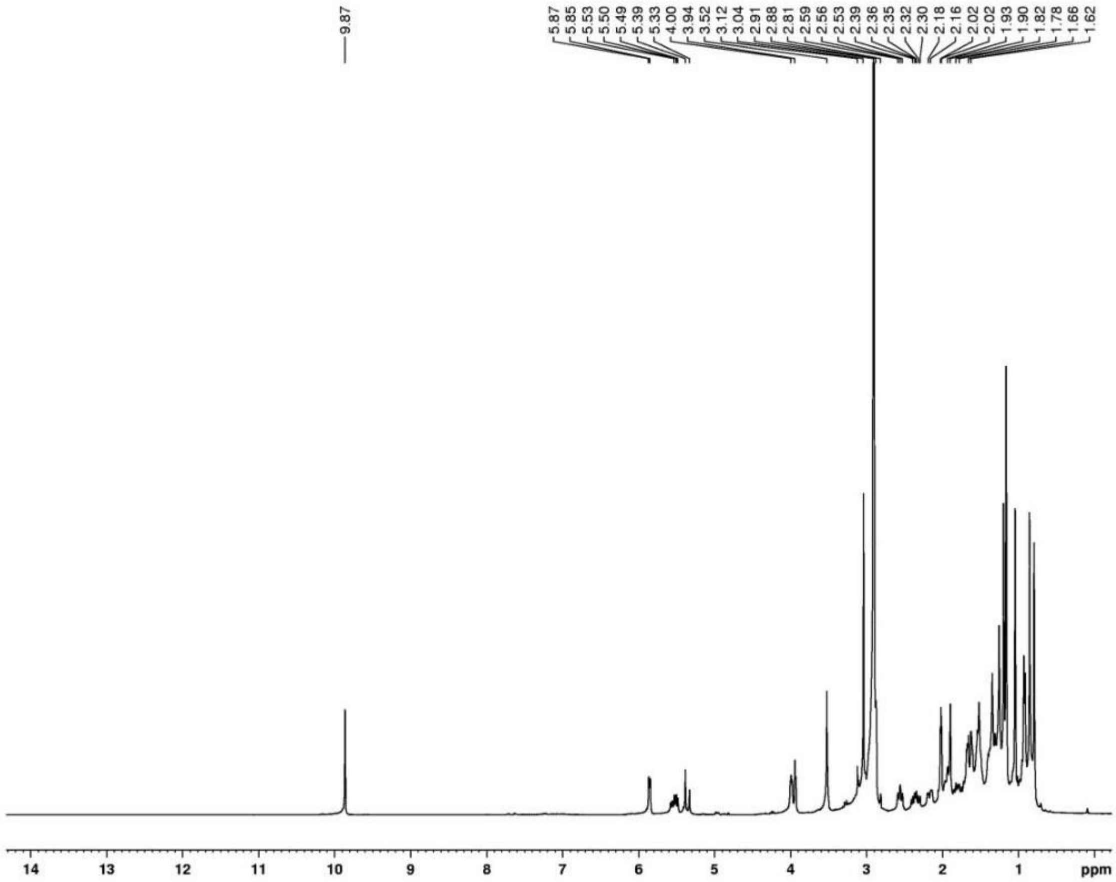

Supplementary Figure 3. ^{13}C NMR spectrum of 25-methoxy-3β, 7β-dihydroxycucurbita-5,23(*E*)-dien-19-al (1) in acetone-d_6 (75 MHz).

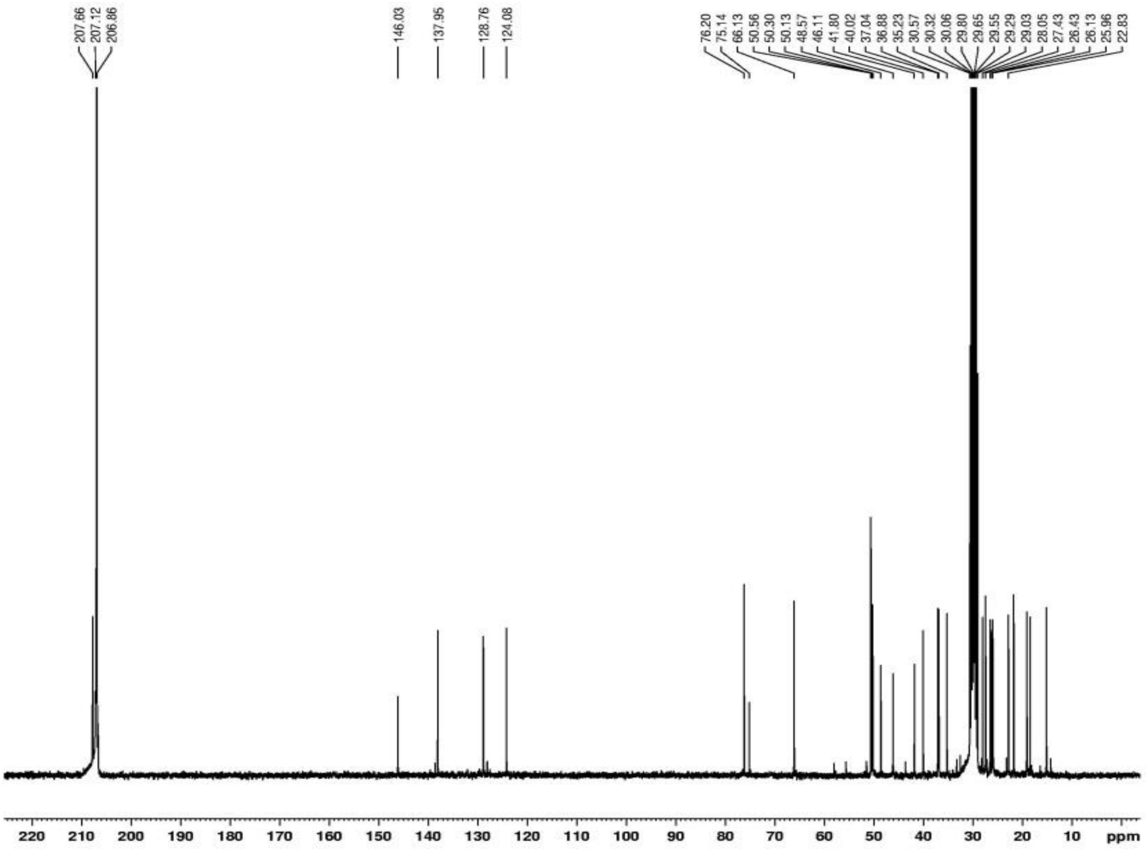

Supplementary Figure 4. ^{1}H NMR spectrum of 3β, 7β 25β-trihydroxycucurbita-5,23(*E*)-dien-19-al (2) in methanol-d_4 (300 MHz).

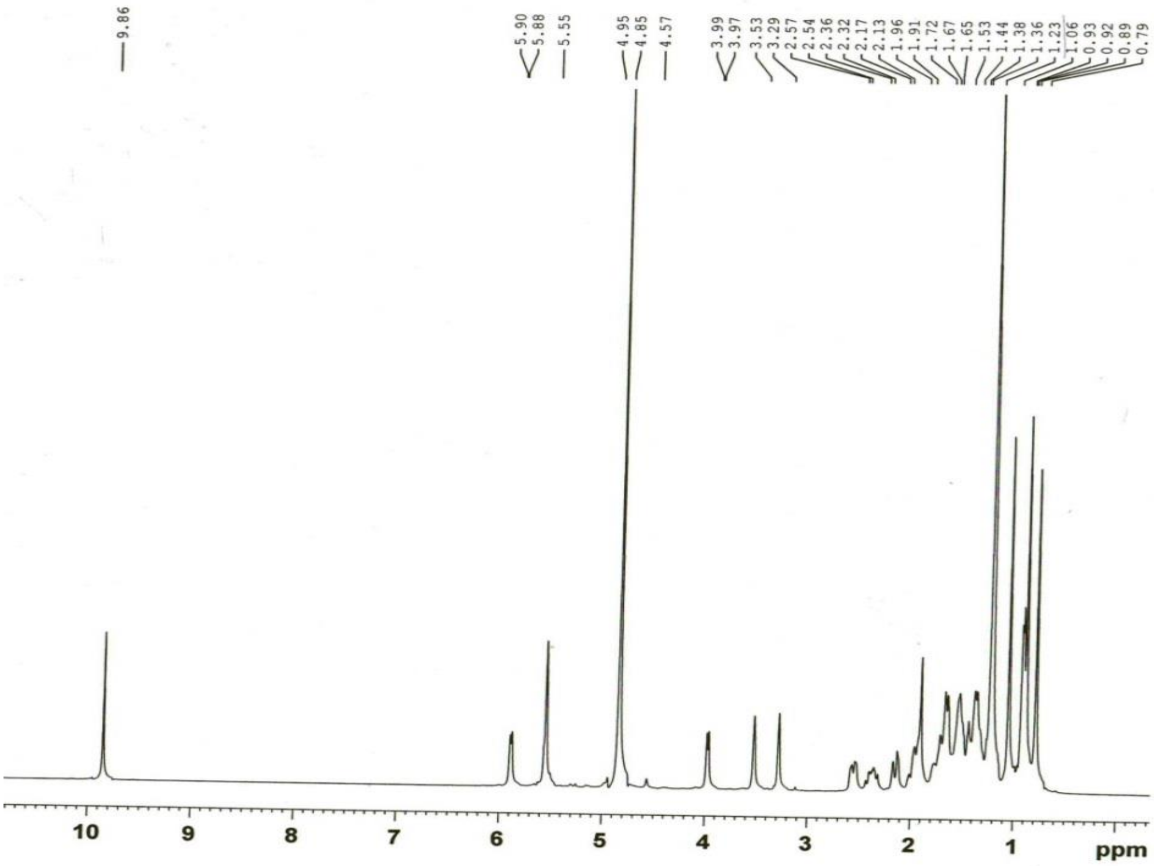

Supplementary Figure 5. ^{13}C NMR spectrum of 3β, 7β, 25β-trihydroxycucurbita-5,23(*E*)-dien-19-al (2) in methanol-d_4 (75 MHz).

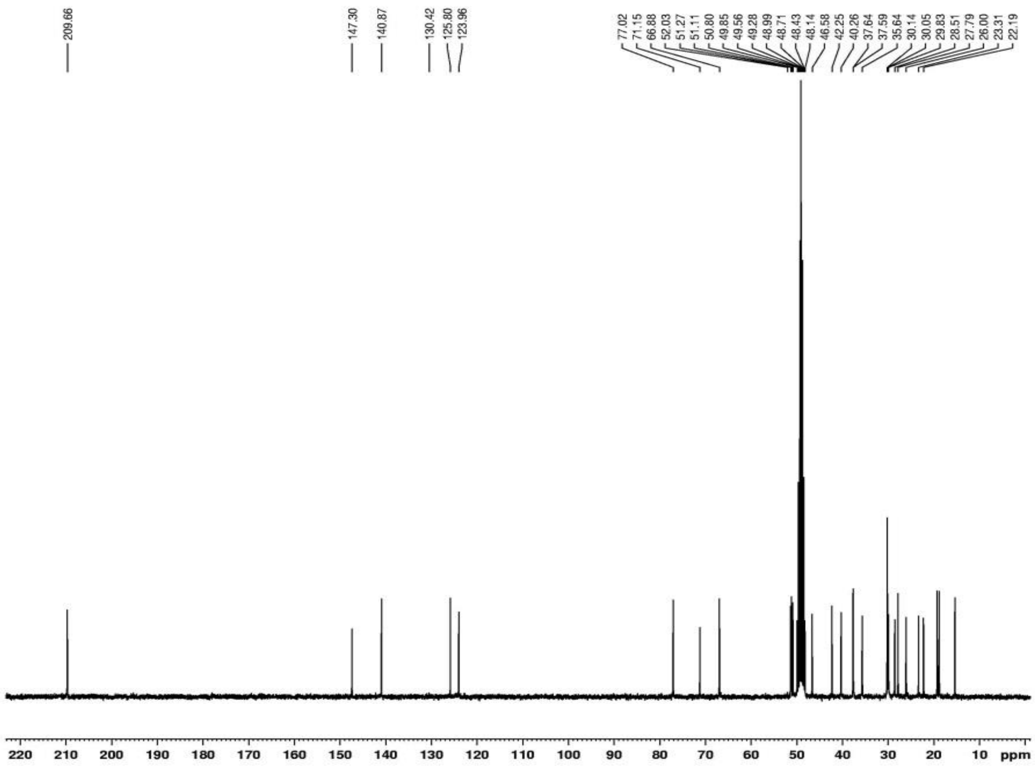

Supplementary Figure 6. ^{1}H NMR spectrum of 19,25-dimethoxy-5β-19-epoxycucurbita-6,23(*E*)-dien-3β-ol (3) in acetone-d_6 (300 MHz).

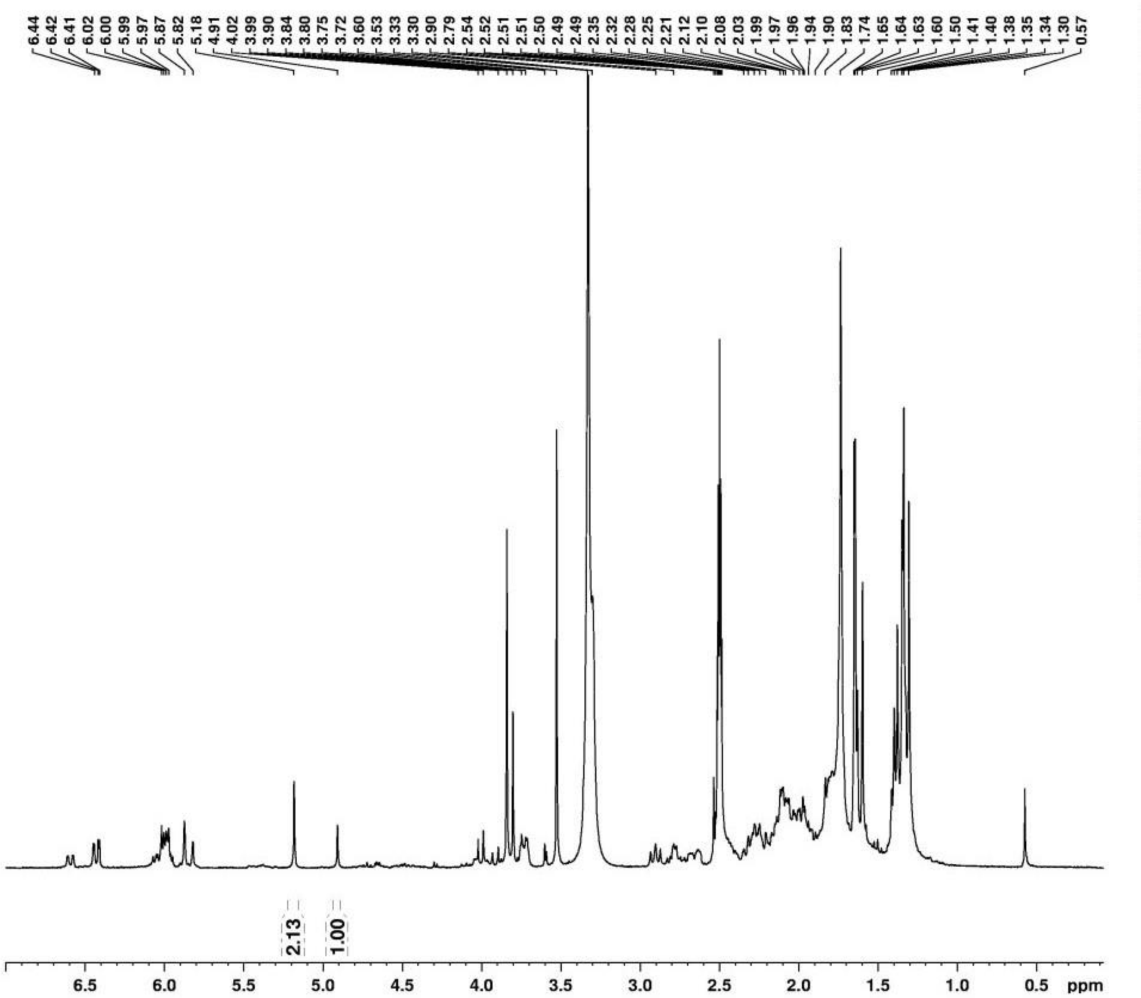

Supplementary Figure 7. ^{13}C NMR spectrum of 19,25-dimethoxy-5β-19-epoxycucurbita-6,23(*E*)-dien-3β-ol (3) in acetone-d_6 (75 MHz).

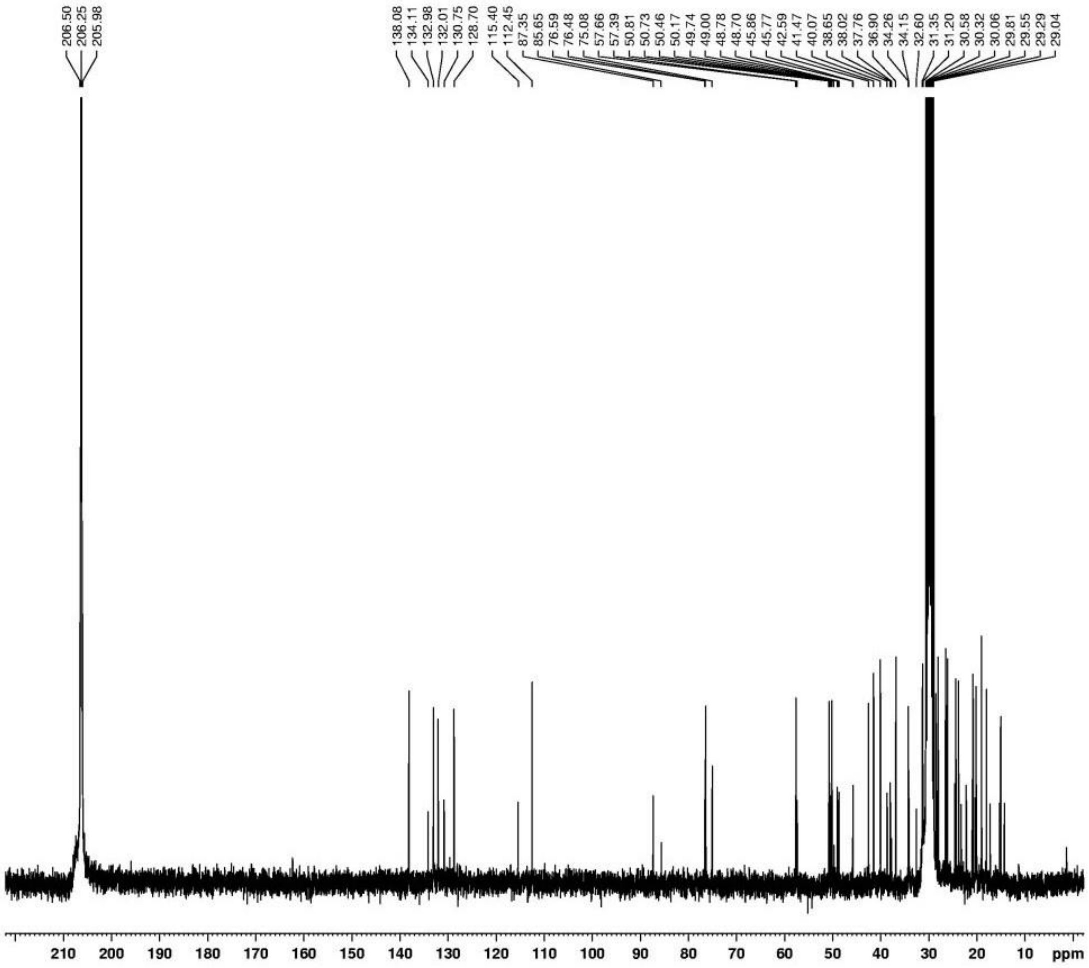

Supplementary Figure 8. ^{1}H NMR spectrum of 19(*R*)-methoxy-5β-19-epoxycucurbita-6,23(*E*)-dien-3β,25-diol (4) in acetone-d_6 (300 MHz).

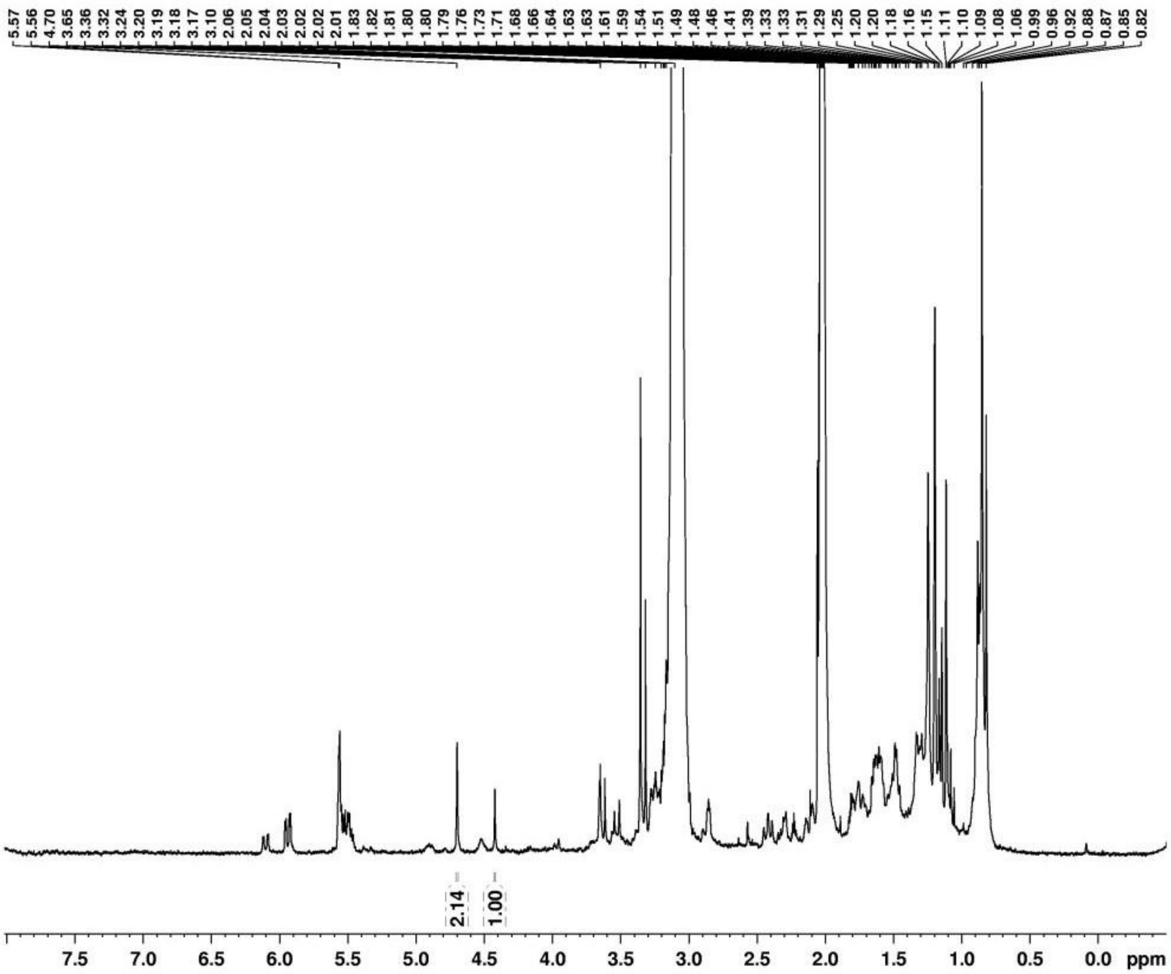

Supplementary Figure 9 ^{13}C NMR spectrum of 19(*R*)-methoxy-5β-19-epoxycucurbita-6,23(*E*)-dien-3β,25-diol (4) in acetone-d_6 (75 MHz) and expansions.

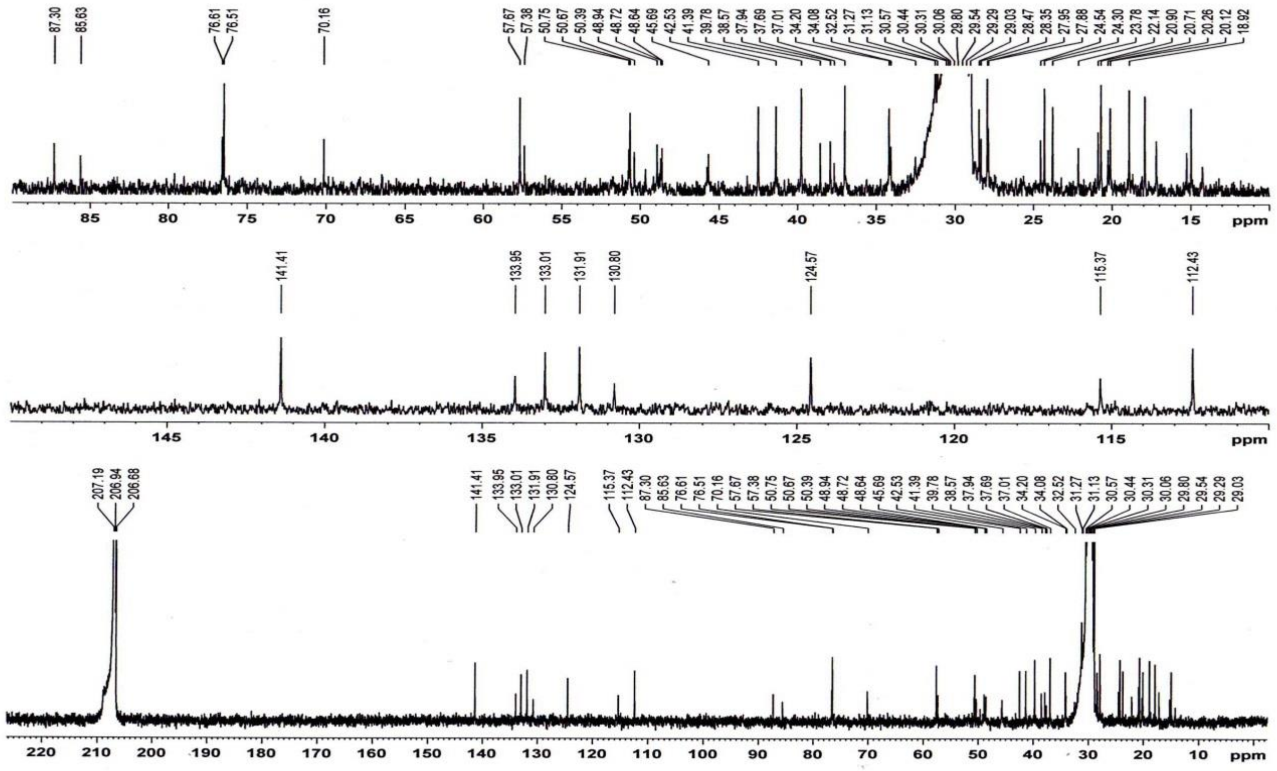

Supplementary Figure 10 HPLC profile of the cucurbitacins 1-2. Stationary phase: Phenomenex Luna® C18 column (5 µm, 100 Å, LC Column 4.6 x 250mm). Isocratic elution with CH_3CN:H_2O (70:30, v/v) and detection at 210 nm.

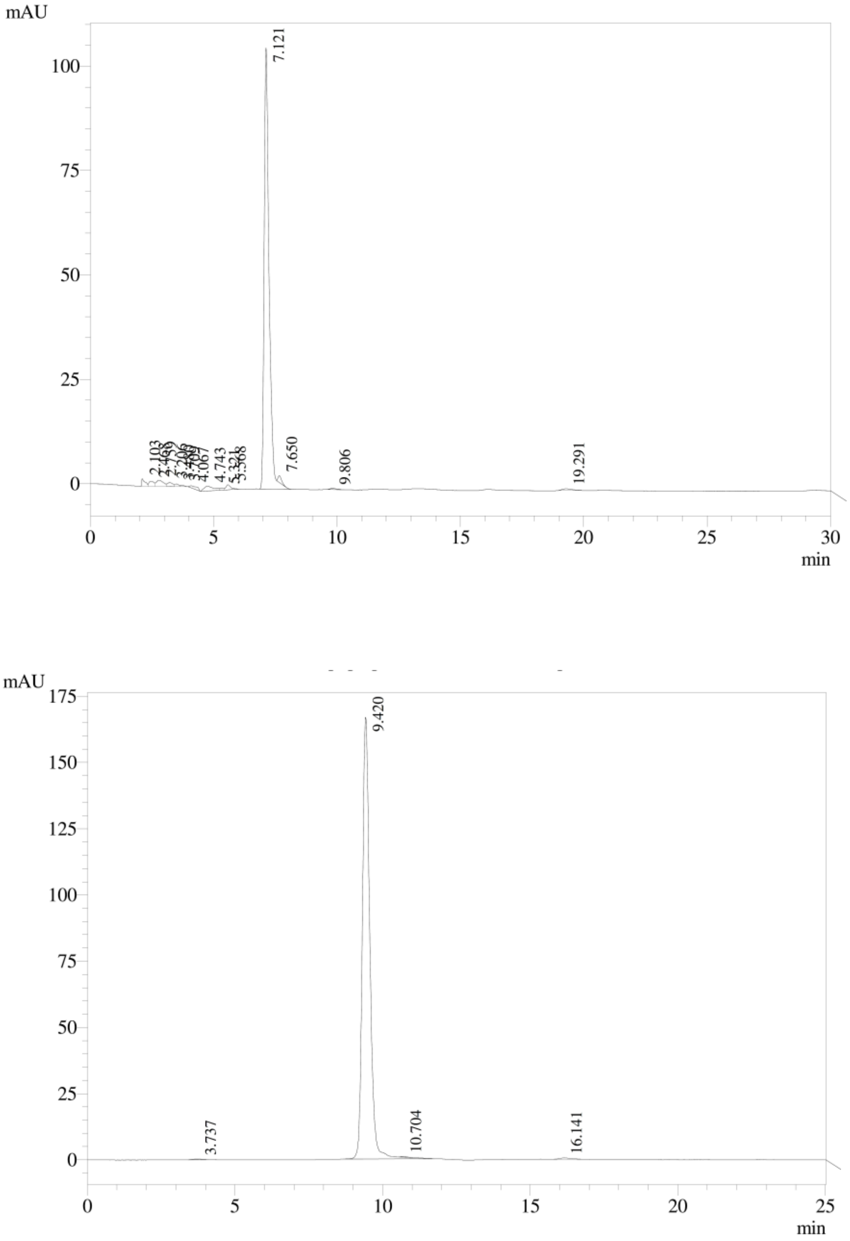

Supplementary Table 1. NMR spectroscopic data of compounds 1-4 (300 MHz for ^{1}H and 75 MHz for ^{13}C), chemical shift (δ_H, δ_C), signal multiplicity and coupling constant (J, Hz).

	**1**[a]		**2**[b]		**3**[a]				**4**[a]			
					R		*S*		*R*		*S*	
	δH	δC	δH	δC	δH	δC	δH	δC	δH	δC	δ	δ
1		21.4		21.17		18.0		17.3		17.9		17.2
2		30.6		30.05		28.1		28.0		27.9		27.9
3	3.12*m*	76.2		77.02		76.5		76.6	3.24*dd* (10.1; 3.1)	76.5	3.32	76.6
4		41.8		42.25		38.0		37.8		37.9		37.7
5		146.0		147.3		87.3		85.6		87.3		85.6
6	5.87*dd* *(3.5)*	124.1	5.89*d* *(4.6)*	123.9	5.97*dd* *(9.7; 2.2)*	133.0	6.13*dd* *(9.7; 2.1)*	134.1	5.94*dd* (8.0;2.2)	133.0	6.10*dd* (9.8;2.1)	133.9
7	4.0d (5.2)	66.1	3.98*d* (5.37)	66.88		132.0		130.8	5.57*dd* (9.8;3.5)	131.9	5.56*dd* (9.8;3.5)	130.8
8		50.6	2.36*s*	46.58	2.90 *dd like*	42.6	1.90 *dd like*	38.7	3.1 *dd like*	42.5	2.06 *dd like*	38.6
9		50.6		51.27		49.0		49.7		48.6		48.7
10	2.56*dd* *(9.2)*	37.0	2.57*m*	37.64		41.5		41.5		41.4		41.34
11		22.8		23.31		23.8		23.3		23.8		22.1
12		29.3		29.83		30.3		30.4		30.6		30.4
13		46.1		42.25		45.8		45.9		45.7		45.7
14		48.6		51.11		48.7		48.8		48.9		48.9
15		35.2		35.64		34.3		34.2		34.2		
16		28.0		26.00		28.5		28.4		28.5		28.3
17		50.1		50.80		50.7		50.8		50.7		50.8
18	0.87s	15.2		15.35		15.0		15.3	0.85 *s*	15.0	0.87 *s*	15.3
19	9.87s	207.6	9.86*s*	209.6	5.18 *s*	112.05	4.91 *s*	115.4	4.70 *s*	112.5	4.42 *s*	115.4
20		36.9		37.59		36.9		36.9		37.0		37.0
21	0.94*d* *(5.0)*	19.0		19.22		19.0		19.1	0.88 *d* *(5.3)*	18.8	0.88 *d*	18.8
22		40.0		40.26		40.0		40.1		39.8		39.8

23	5.5*ddd* *(5.5;9.0;15.7)*	128.8	5.85; 5.56	125.8	5.48* *m*	128.7		128.7	5.59* *m*	124.6	5.59 *m*	124.6
24	5.39*dd* *(15.8; 1.1)*	137.9	5.72; 5.55	140.8	5.38*d* *(15.9)*	138.1		138.1	5.58* *m*	141.4	5.58 *m*	141.4
25		75.1		71.15		75.1		75.1		70.2		70.2
26	1.19*s*	26.1		30.05		26.2		26.2	1.59;1.0 *s*	31.3	1.59;1.0 *s*	31.1
27	1.19*s*	26.4		30.05		26.5		26.5	1.59;1.0 *s*	31.3	1.59;1.0 *s*	31.1
28	1.27*s*	27.4		27.79		20.2		20.3	1.11 *s*	20.7	1.15 *s*	20.9
29	1.06*s*	26.0		28.51		24.2		24.6	0.82 *s*	24.3	0.85 *s*	24.5
30	0.81*s*	18.4		18.78		20.8		21.0	0.87 *s*	20.1	0.87 *s*	20.3
19-OCH_3	-	-	-	-	3.30 *s*	57.7	3.33 *s*	57.4	3.36 *s*	57.7	3.32 *s*	57.4
31-OCH_3	3.52*s*	50.3	-	-	2.90 *s*	50.2	2.90 *s*	50.4				

[a] data recorded in acetone-d_6; [b] data recorded in CD_3OD

* Overlapped signals

Notes: *d* - doublet; *dd* - double of doublets; *ddd* - doublet of doublet of doublets; *m* - multiplet; *s* - singlet;
